# Exploring the biodiversity of plant proteins for sustainable foods: Composition and emulsifying properties of the proteins recovered by aqueous extraction from camelina (*Camelina sativa* L.) seeds

**DOI:** 10.1016/j.crfs.2024.100922

**Published:** 2024-11-10

**Authors:** Christelle Lopez, Hanitra Rabesona, Valérie Beaumal, Hélène Sotin, Bruno Novales, Marc Anton

**Affiliations:** a INRAE, UR BIA, F-44316, Nantes, France; b INRAE, UR BIA, F-35653, Le Rheu, France; c INRAE, PROBE Research Infrastructure, BIBS Facility, F-44316, Nantes, France

**Keywords:** Plant protein emulsifier, Plant-based food, Oilseed, Oil-in-water emulsion, Sustainable food, Sustainable crop

## Abstract

The food transition towards an increased consumption of plant proteins aimed at limiting environmental impacts requires a diversification of plant protein sources. In this study, we explored the potentialities of the sustainable oilseed crop camelina to provide dietary proteins and to prepare oil-in-water emulsions. An innovative green refinery process, including the removal by ultrasound of the mucilage attached at the surface of the seeds and extraction by grinding in water at pH 8, was used to recover aqueous extracts containing camelina seed proteins. These proteins, mainly composed of the 11S globulin cruciferins and the 2S albumin napins, contained the nine essential amino acids of nutritional interest. Camelina seed proteins exhibited low solubility in the range of pH 5.5–2, attributed to the cruciferins. Oil-in-water emulsions stabilized by camelina seed proteins revealed the preferential adsorption of napin at the oil/water interface and exhibited physical instability below pH 7. Homogenization of the whole aqueous extracts containing oil bodies (OBs) induced the adsorption of cruciferins and napins together with oleosins at the surface of homogenized OBs, and their sedimentation below pH 6.5. Camelina seed proteins adsorbed at the oil/water interface governed the surface properties of the oil droplets and homogenized OBs as well as the physical stability of the emulsions as a function of pH. This work brings new insights into the potentialities of camelina seed proteins recovered by aqueous extraction that may serve as natural plant-based functional ingredients in the food industry and that will contribute to the development of innovative and sustainable plant-based food emulsions.

## Highlights

• The biodiversity of plant-based proteins needs to be explored for sustainable food emulsions.
• Camelina seed proteins can be recovered using a green refinery process based on aqueous extraction.
• Camelina seed proteins have excellent emulsifying properties.
• The physical stability of camelina seed proteins and O/W emulsions is altered below pH 6.
• Plant proteins from sustainable crops such as camelina can be used as natural emulsifiers.

## Graphical abstract

**Figure:**
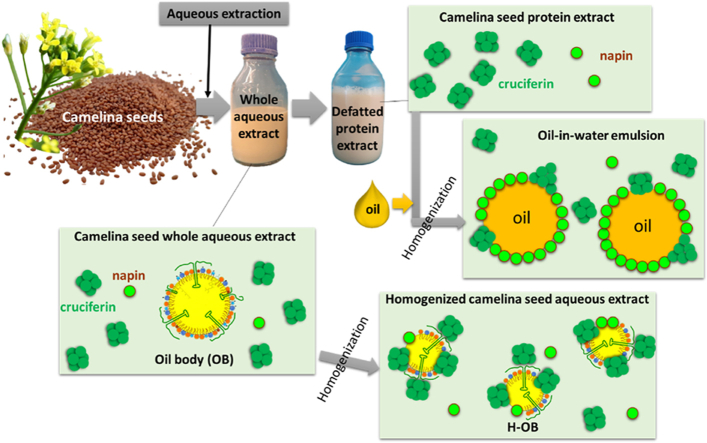

## 1 Introduction

It has become necessary to explore new protein sources for the human diet to respond to the increasing population worldwide and its associated environmental impacts. Furthermore, there is a growing interest in gradually replacing animal-based proteins (i.e., from meat, eggs, milk and dairy products) with plant-based proteins in the human diet to mitigate greenhouse gas emissions and to reduce the carbon footprint of foods (McClements and Grossmann, 2021). This dietary protein transition requires a large amount of available plant proteins from sustainable crops to feed the world population, as well as a diversification of plant-based protein sources. In this context, investigating the biodiversity of oilseeds as potential new sources of plant-based proteins, and exploring the nutritional and functional challenges faced by protein diversification, could be an innovative way to efficiently contribute to sustainability for the planet. Moreover, there is an increasing demand of consumers for minimally processed protein-rich and plant-based foods associated with health-promoting properties, including plant proteins of high nutritional quality containing essential amino acids (EAAs) and plant lipids rich in polyunsaturated fatty acids (PUFAs).

There is currently a growing interest in camelina (*Camelina sativa* L.), an annual sustainable oilseed crop belonging to the Brassicacea*e* family (Arshad et al., 2022; Sydor et al., 2022). Camelina cultivation is currently low but has increased in recent years, especially with production in North America, Russia as well as Central and Eastern Europe (Matteo et al., 2023). However, it is garnering renewed attention due to environmental attributes and agronomic qualities, e.g., adaptability to diverse environmental conditions and low agricultural inputs (i.e., water, pesticides and nutrients), compared to other traditional oilseed crops, in addition to a relatively strong resistance to insects, pests and microbial diseases (Arshad et al., 2022; Ghidoli et al., 2023; Matteo et al., 2023; Neupane et al., 2022; Veljković et al., 2022). Moreover, camelina is significantly compatible with existing farming practices, is characterized by a very short crop cycle, and can be easily grown on poor and marginal lands. Camelina is a climate-resilient oilseed crop that differs from other traditional oilseed crops. For these reasons, camelina is suitable for less favored areas and represents a valuable resource to fight and to adapt agricultural systems to climate change. Camelina seeds, oil and seed meal or cakes are of special interest for various industries, including the energy (biofuel and biodiesel production) and food industries (ingredients for functional foods). They also have potential for other uses, e.g., oleochemicals in non-food industries such as cosmetics, bio-based lubricants or surfactants and cleaning agents on the worldwide market in search of greener alternatives to be used as feedstock or to be substituted for petroleum-derived oil to offset greenhouse gas emissions (Berti et al., 2016; Clavijo-Bernal et al., 2024; Matteo et al., 2023).

Camelina oilseeds are of great nutritional interest due to their high amount of lipids (27–49%) and their richness in essential ω3 PUFAs, proteins (24–31%), dietary fiber, carbohydrates, vitamins, minerals and other bioactive compounds (plant sterols), including natural antioxidants (phenolic compounds, tocopherols) (Arshad et al., 2022; Neupane et al., 2022). Camelina oil contains 50.8–66.6% PUFAs, of which 31–40% are α-linolenic acid (ω3 PUFA) (Abad and Shahidi, 2021; Lopez et al., 2023b; Ostrikov et al., 2021). These essential ω3 PUFAs contribute to the interest in camelina oil's benefits for human nutrition and health as it has recently been linked to the reduction of blood serum cholesterol levels and the improvement of serum lipid profiles (Karvonen et al., 2002; Manninen et al., 2019). The ω3 PUFA-rich oilseeds are therefore promising sources of plant nutrients for human consumption and are of special interest to correct low ω3 dietary intake as well as ω6/ω3 balance. Among oilseeds containing interesting fatty acid profiles, camelina is identified as one of the main candidates for the future European bioeconomy (Righini et al., 2016; Zanetti et al., 2021). Camelina seeds are mostly used for non-food applications. They have limited food applications, mainly cold-pressed oil or seeds used as food ingredients (Mondor and Hernández-Álvarez, 2022; Neupane et al., 2022). Despite the interesting chemical composition of camelina seeds, few research studies have been performed to explore their potential in food applications such as the valorization of camelina seed oil bodies (OBs, the natural oil droplets found in the seeds also called oleosomes) as a natural oil-in-water emulsion (Lopez et al., 2023b).

Camelina seed processing at this time mainly consists of the mechanical warm or cold pressing of whole oilseeds. Pressing can eventually be followed by solvent (i.e., n-hexane) or supercritical carbon dioxide extraction of the remaining oil in the press cake. This conventional processing method has been widely used for oil extraction and recovery (Berti et al., 2016; Veljković et al., 2022). However, the solvent residue left in the oil and in the meal poses a health risk. After oil extraction, the residual defatted seed press cake, also referred to as seed meal, is still rich in high-value nutritional compounds (Tavarini et al., 2021). This by-product of the camelina oil extraction process typically contains up to 40% crude protein and 10–15% residual oil that can induce oxidation and off-flavors (Li et al., 2014). Camelina meal is used for protein extraction (i.e., alkaline or salt extraction) and protein fractionation (i.e., at acidic pHs) to produce plant protein ingredients, i.e., concentrates and isolates (Boyle et al., 2018; Matteo et al., 2023; Ngo and Shahidi, 2021; Wanasundara, 2011). The detrimental effects of solvent extraction on the denaturation of camelina seed proteins upon processing from defatted seed meal could impact their functional properties (e.g., solubility, heat induced gelation, emulsifying and foaming properties), their nutritional properties including digestibility and bioavailability, as well as their allergenicity. Other green refinery strategies therefore need to be developed to preserve the functional and nutritional qualities of camelina seed proteins.

Over the last 10 years, there has been a growing interest in developing innovative refinery processes, integrated and green (organic and toxic-solvent free), capable of preparing aqueous extracts and recovering natural OBs from oilseeds (e.g., rapeseed, sunflower, linseed, hemp, chia, camelina) and nuts (e.g., walnut) of nutritional interest for food applications (Lopez et al., 2021, 2023b, 2023a; Nikiforidis, 2019). Integrated processes including soaking of the seeds and grinding at alkaline pHs have been successfully used to co-extract and recover OBs along with proteins by aqueous extraction from oilseeds (Acevedo-Fani et al., 2020; Garcia et al., 2021; Nikiforidis et al., 2014; Nikiforidis and Kiosseoglou, 2009). After removal of OBs by centrifugation, the by-products of these green refinery processes are defatted aqueous extracts containing seed proteins.

Aqueous extraction of camelina seed proteins could be an alternative process, avoiding the denaturation of the proteins generally observed during solvent-assisted mechanical pressing of the seeds. However, camelina seeds are mucilagenous seeds, meaning that they produce and expel high-viscosity mucilage when placed in contact with water. The oilseed mucilage has been the subject of several studies dedicated to extraction processes, characterization and valorization as an ingredient that has high potential as a natural thickener, stabilizer and emulsifier for food, cosmetic, and pharmaceutical applications (Campos et al., 2016). The efficient recovery of OBs and proteins from camelina seeds requires the removal of mucilage before aqueous extraction, e.g., using ultrasound (Fabre et al., 2020; Lopez et al., 2023b).

Food formulations cover a range of pH values from basic to acidic. Some food products require a pH of 5.5 or lower for microbial stability, or to reach gelification (e.g., in dairy protein based-gels and dairy analogues). Examining the physical stability of plant-based proteins and plant-protein stabilized emulsions as a function of pH is therefore necessary. In addition, increasing our knowledge about the physical stability of plant proteins and emulsions as a function of pH is important to better understand their behavior in the gastro-intestinal tract upon digestion, particularly in the acidic conditions of the gastric phase. The properties and physical stability as a function of pH of camelina seed proteins and emulsions prepared with these proteins are poorly known at this time.

Paving the way for the use of camelina seed proteins recovered by aqueous extraction will contribute to plant-based protein diversification and to innovative food applications. The objectives of this study were therefore: (i) to produce whole aqueous extracts from camelina oilseeds using the green refinery process previously developed (Lopez et al., 2023b); (ii) to characterize the composition and properties of the camelina seed proteins recovered by aqueous extraction; (iii) to prepare oil-in-water emulsions stabilized by camelina seed proteins and examine their physical stability as a function of pH; and (iv) to investigate the role of interfacial proteins on the physical stability as a function of the pH of homogenized camelina seed whole aqueous extracts containing oil bodies. A multiscale approach permitted the recovery of scientific information from the camelina seed proteins to the emulsions as a function of pH, and from the macroscale (physical stability of the proteins and emulsions) to the microscopic level (confocal microscopy observations) and to the level of the particles by determining the surface properties of the proteins and the oil droplets (analysis of interfacial protein composition and zeta-potential measurements).

## 2 Materials and methods

### 2.1 Materials

**Camelina oilseeds.***Camelina sativa,* cultivar “Celine” variety, was used to provide Camelina (*Camelina sativa* L.) oilseeds. The oilseeds were grown at INRAE (Versailles, France) in open fields during spring 2019. They were maintained at 4 °C for long-term storage.

**Camelina seed oil.** Organic camelina seed oil (*huile vierge bio cameline*) was produced by the Huilerie Vigean (36 Clion-sur-Indre, France) and purchased at the Biocoop store (29 Landivisiau, France).

### 2.2 Camelina seed processing

**Fig. 1:**
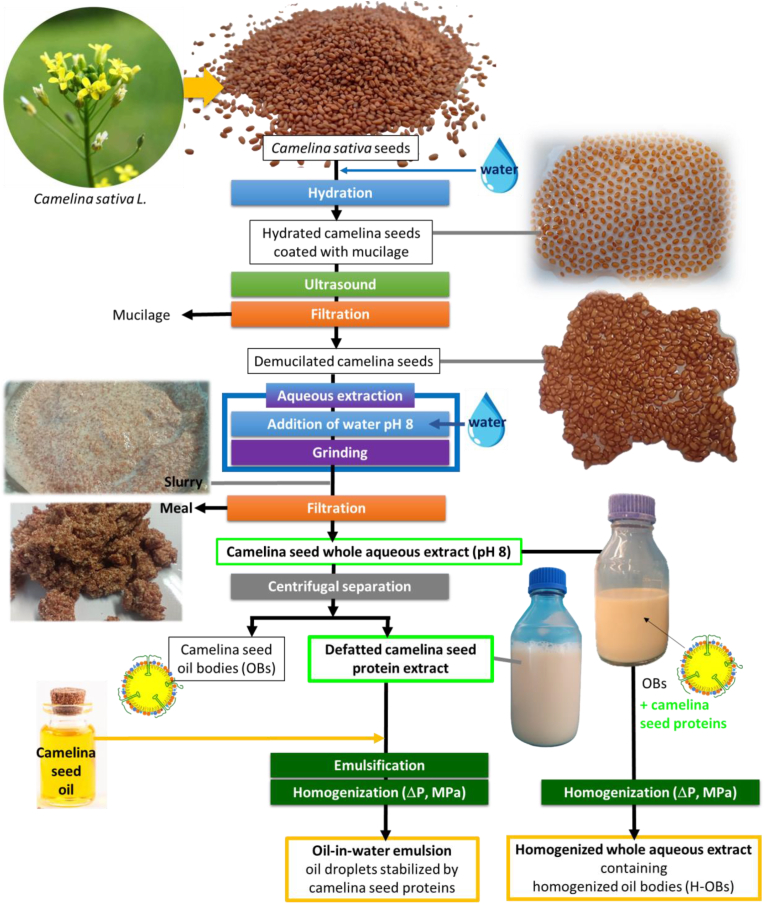
Diagram of the refinery process used for the preparation of whole aqueous extracts and defatted protein extracts from camelina seeds. Images are introduced to visualize camelina seeds (top), hydrated camelina seeds coated with mucilage (right), mucilage-free camelina seeds obtained after ultrasonication and removal of the mucilage (right), the slurry (left), the meal recovered after filtration of the slurry (left), whole aqueous extracts and the defatted aqueous extracts containing the proteins. The bottom part of this schematic diagram presents the preparation of the oil-in-water emulsions stabilized by the camelina seed proteins, and the preparation of homogenized whole aqueous extracts containing oil bodies.

The preparation of aqueous extracts from camelina seeds is presented in Fig. 1. This refinery process involved environmentally-friendly techniques (absence of organic solvent). The first step consisted in the hydration of the camelina seeds. Camelina seeds (batches of 200 g) were soaked in ultrapure water (1:5 w/v), adjusted at pH 8.0 (using 2 M NaOH), and stirred for 12 h at 4 °C. The second step consisted in the removal of the mucilage surrounding the seeds using ultrasound, as detailed in (Lopez et al., 2023b) and adapted from (Fabre et al., 2020). The other steps of the refinery process were similar to the aqueous extraction process previously developed for hemp seeds, chia seeds and camelina seeds (Lopez et al., 2021, 2023b). Grinding of the demucilated camelina seeds was performed in water adjusted to pH 8.0 to promote protein solubilization (grinding parameters: 3 min at a speed of 6000 rpm; Turbomix plus, Moulinex, France). The aqueous extracts were recovered from camelina seeds, as previously reported (Lopez et al., 2023b). The resulting slurry (see picture, Fig. 1) was filtered through a layer of cheesecloth. The external part of the seeds (i.e., shells, solid residues) and cell wall components (see picture of the meal, Fig. 1) were removed. The filtrates that contained the camelina seed OBs and proteins as well as the fibers and phenolic compounds corresponded to the camelina seed whole aqueous extracts. Camelina seed whole aqueous extracts were centrifuged at 7000 *g* for 30 min at 15 °C (Eppendorf® 5810 R centrifuge; Merck KGaA, Darmstadt, Germany). The layer of OB-rich cream at the top of the tubes was manually collected. This step was repeated twice. The defatted aqueous extracts (fat content below 0.1 g/100 g) were considered as camelina seed protein extracts and were used for experiments without any further purification of the proteins. To prevent the growth of bacteria, sodium azide (NaN_3_, 0.01 %wt) was added to the whole aqueous extracts and to the defatted protein extracts.

### 2.3 Camelina seed proteins: content and composition in amino acids

#### 2.3.1 Protein content

The protein content of camelina seeds was determined by the AOAC standard using the Kjeldahl method. The total protein contents recovered in the defatted camelina seed aqueous extracts were determined by the Dumas combustion analysis method (Elementar, Langenselbold, Germany). A nitrogen-to-protein conversion factor of 5.7 was used (Ntone et al., 2021).

#### 2.3.2 Amino acid analysis

The total amino acid concentrations were determined in the camelina seeds and in the defatted aqueous extracts (n = 3 independent aqueous extractions) after total acid hydrolysis of the samples in 6 N HCl for 24 h at 110 °C. The hydrolyzed samples were adjusted to pH 2.2 using NaOH. The samples were analyzed by high performance liquid chromatography (HPLC) equipped with a sodium oxidized column and cation exchange resin. Post-column derivatization of the amino acids to ninhydrin and spectrophotometric detection at 570 nm was then performed, except for proline, which was detected at 440 nm. A replicate of each sample was oxidized before acid hydrolysis by performic acid and incubated at 4 °C for 16 h to quantify the sulfur amino acids (methionine and cysteine). To determine the tryptophan content, the samples were hydrolyzed in alcaline conditions using octa-hydrated barium hydroxide in an autoclave at 110 °C for 16 h. Before hydrolysis, 5-methyl-tryptophan was added as an internal standard. The tryptophan content was determined by reverse-phase HPLC analysis.

### 2.4 Solubility profile of camelina seed proteins as a function of pH

The camelina seed protein solubility profile was evaluated in water at pHs ranging from 1 to 12. First, 25 g of a defatted protein aqueous extract were adjusted at different pHs (denoted pHx with x ranging from 1 to 12) and were magnetically stirred for 1 h at 20 °C. The pH was checked and adjusted if required. Each sample at pHx was distributed into two tubes: a first tube (10 g) to determine the total protein content in the sample at pHx, and a second tube (15 g) that was centrifuged at 10,000 g for 20 min at 4 °C to recover the soluble proteins in the supernatant. The protein solubility experiments were performed with three independent defatted protein extracts recovered from camelina seeds. The total nitrogen contents in the sample at pHx and in the corresponding supernatant at pHx were determined by the Dumas combustion analysis method (Elementar, Langenselbold, Germany). The nitrogen-to-protein conversion factor of 5.7 was used (Ntone et al., 2021). The protein solubility (PS, %) was calculated as the ratio between the protein content in the supernatant at a specific pHx and the total protein content in the sample at pHx. The PS formula is as follows:

$$\text{PS}\;\left(\%\right)=\frac{\text{protein}\;\text{content}\;\text{in}\;\text{the}\;\text{supernatant}\;\text{at}\;a\;\text{specific}\;\text{pHx}\;}{\text{total}\;\text{protein}\;\text{content}\;\text{of}\;\text{the}\;\text{whole}\;\text{sample}\;\text{at}\;\text{pHx}}\;x\;100$$

### 2.5 Preparation of oil-in-water emulsions and homogenized whole aqueous extracts

Oil-in-water emulsions were prepared with the defatted camelina seed protein aqueous extracts (pH 8.0) recovered after centrifugation of the whole aqueous extract and removal of the OBs (Fig. 1). The amount of camelina seed oil necessary to prepare a 3 %wt oil emulsion was mixed with the protein extract (e.g., for 200 g emulsion: 6 g oil, weight adjusted to 200 g with the protein aqueous extract). The emulsions were prepared using a multi-step process including emulsification by high-shear mixing with a Polytron (25,000 rpm, 30 s), followed by the use of a laboratory high-pressure homogenizer (PandaPlus 1000, GEA Niro Soavi, Italy). Homogenization was performed at pressures varying from 3.5 to 50 MPa (Fig. 1).

Whole camelina seed aqueous extracts containing the OBs were homogenized at 20, 35 and 60 MPa using a laboratory high-pressure homogenizer (PandaPlus 1000) (Fig. 1).

### 2.6 Determination of the protein profiles by gel electrophoresis

The protein profiles in the camelina seed aqueous extracts and the interfacial protein composition of emulsion lipid droplets, oil bodies (OBs) and homogenized OBs were determined by sodium dodecyl sulfate polyacrylamide gel electrophoresis (SDS-PAGE).

#### 2.6.1 Preparation of the oil droplets

Washing of emulsion oil droplets and camelina seed OBs was performed to remove non-adsorbed proteins from the O/W interface. The emulsion (5 g) was mixed with a solution (5 g) containing 50 %wt saccharose. Then, in 45 mL plastic centrifuge tubes, 10 mL of the treated emulsion were deposited under 30 mL of a solution containing 5 %wt saccharose. The tubes were centrifuged with adapted parameters as a function of the size of the oil droplets to form a layer of washed oil droplets. The tubes were centrifuged for 20 min at 3000 *g* (15 °C), except for the homogenized whole aqueous extracts (35 MPa and 60 MPa) and the emulsion prepared at 50 MPa that were centrifuged for 20 min at 6000 *g* (15 °C). The washed emulsion oil droplets and OBs recovered at the top of the tubes were used for SDS-PAGE analysis.

#### 2.6.2 Gel electrophoresis

The protein profiles of the samples were characterized by SDS-PAGE. Gradient 4–15% Mini-Protean TGX Precast Gels were used with the Mini-Protean Tetra Cell system (Bio-Rad Life Science, France). All the reagents were purchased from Bio-Rad Life Science (France). The protein profiles of the samples were examined in denaturing and either reducing or non-reducing conditions. For the denaturing and reducing conditions, the samples were diluted in 2x Laemmli denaturing sample buffer with β-mercaptoethanol 5% (referred to as + BME; a reducing agent that breaks down the disulfide bond linkages). For the denaturing and non-reducing conditions, the samples were diluted in 2x Laemmli denaturing sample buffer without BME (referred to as –BME). The migration buffer was composed as follows: 25 mM Tris, 192 mM glycine, 0.1% SDS, according to the protocol described by (Laemmli, 1970). The samples were heated for 5 min at 100 °C. Each protein sample was then loaded on a sample well of the 4–15% gradient gel. For molecular weight (MW) calibration, MW protein markers from 10 to 250 kDa (Precision Plus Protein Standards, All Blue, Bio-Rad Life Science, France) and from 14.4 to 116 kDa (unstained molecular weight marker; Euromedex, Souffelweyersheim, France) were used. The migration of the proteins was performed at 200 V for 45 min. Each gel was stained with Coomassie Brilliant Blue G-250 staining solution under gentle agitation for 2 h, according to the method used by Lawrence and Besir (2009). Each gel was then rinsed with distilled water. The gels were then scanned on a flatbed scanner (Image Scanner iii; GE Healthcare Europe, Velizy- Villacoublay, France).

### 2.7 Physical stability of the samples as a function of pH

The physical stability as a function of pH of the defatted camelina seed protein extracts, oil-in-water emulsions and homogenized whole aqueous extracts was evaluated at the macroscopic scale. The samples were adjusted at various pH values using HCl or NaOH solutions. The samples were stored in 15-mL plastic tubes, stored at room temperature (19 ± 1 °C) for 4 h, at 4 °C overnight, and then centrifuged (1500 *g*, 15 min, 15 °C) to accelerate the physical destabilization observed in the tubes. Pictures of the tubes were taken to characterize the physical stability of the samples as a function of pH.

### 2.8 Particle size measurements

The size distributions of emulsion oil droplets, OBs and homogenized OBs were determined by using a laser diffraction analyzer (Horiba LA-960V2; Retsch Technology, Germany). The refractive indices used were 1.33 for the continuous phase (water) and 1.47 for the camelina oil, as previously reported (Lopez et al., 2023b). The samples were characterized in water and after 10-fold dilution in sodium dodecyl sulfate (SDS: 1 %wt). SDS disrupts flocculation between oil droplets and permits the determination of the size distribution of individual oil droplets. The samples were added as appropriate in ultrapure water for the particle size measurements. Each sample was analyzed in triplicate at room temperature.

### 2.9 Zeta potential measurements

The zeta-potential (ζ-potential) experiments were performed with a Zetasizer Nano ZS (Malvern, Germany). The samples of defatted protein aqueous extracts, oil-in-water emulsions and homogenized whole aqueous extracts were diluted in water (50 μl sample in 20 mL water). The samples were then adjusted at various pHs using NaOH or HCl solutions, as previously reported for camelina seed OBs (Lopez et al., 2023b). One mL of the diluted samples was placed in a ζ-potential specific cuvette. Each cuvette was placed in the chamber of the Zetasizer Nano ZS apparatus and equilibrated for 5 min at 20 °C before the measurements that were run five times at 20 °C for each sample. The ζ-potential values were calculated from the electrophoretic mobility of the proteins or oil droplets according to the Smoluchowski approximation and Henry's law. The measurements were performed on at least three independent samples.

### 2.10 Microstructure: confocal laser scanning microscopy

The microstructures of the samples (i.e., defatted camelina seed protein aqueous extracts, oil-in-water emulsions and homogenized camelina seed whole aqueous extracts at pH 8 and as a function of pH) were examined by confocal laser scanning microscopy (CLSM). A NIKON A1R microscope (NIKON, Champigny sur Marne, France) was used. A x60 oil immersion objective was used for the observations. The proteins were stained with the fluorescent dye Fast Green FCF (10 mg/mL in water; Sigma-Aldrich, St. Louis, MO, USA; excitation wavelength = 636 nm). The internal cores of the emulsion oil droplets, OBs and homogenized OBs were stained with the fluorescent lipid-soluble Nile Red dye (100 μg/mL in propanediol; Sigma Aldrich, St Louis, MO, USA; excitation wavelength = 560 nm). Prior to the microscopic observations, the samples were kept for at least 20 min at room temperature.

### 2.11 Statistical analysis

The analyses performed to determine the amino acid compositions of the seeds and protein extracts were repeated independently at least three times. The results are presented as mean values and standard deviations. The General Linear Model procedure of Statgraphics Plus, version 5 (Statistical Graphics Corp., Englewood Cliffs, NJ, USA) was used to perform analyses of variance (ANOVA). Differences between the treatment means were compared at the 5% level of significance using Fisher's least significance difference (LSD) test.

## 3 Results and discussion

The camelina seeds contained 22.4 ± 0.1 g of proteins per 100 g. This value is lower than values reported in the literature, i.e., 24–31 g/100 g (Arshad et al., 2022). The amount of lipids in the camelina seeds was 42.1 ± 1.6 g per 100 g, in agreement with previous reports (Arshad et al., 2022; Lopez et al., 2023a; Neupane et al., 2022).

### 3.1 Camelina seed proteins recovered by aqueous extraction: amino acid (AA) composition and protein profile

**Fig. 2:**
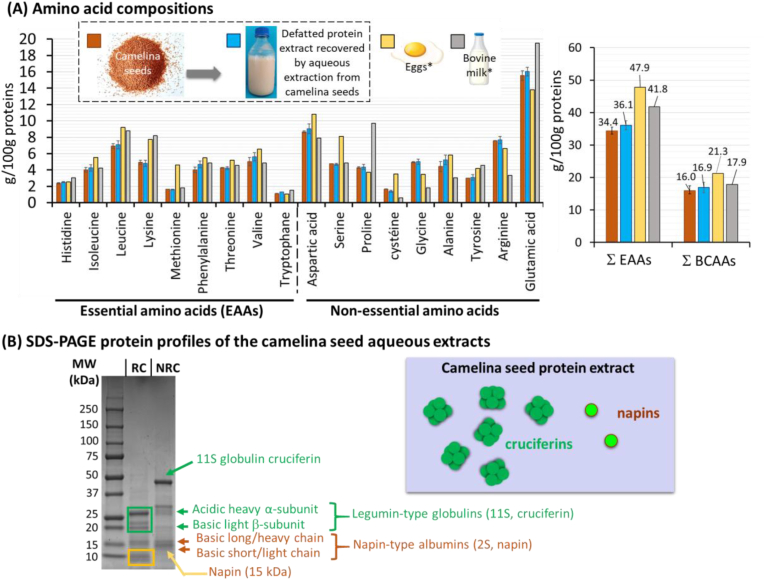
Amino acid and protein compositions. **(A)** Left: amino acid compositions (expressed in g/100 g proteins) of *camelina sativa* L. seeds, defatted protein aqueous extracts recovered from camelina seeds (n = 3), and the comparison with the amino acid compositions of eggs (∗ data calculated from (Dajnowska et al., 2023)) and bovine milk (∗ data calculated from (Claeys et al., 2014)); Right: sum of the essential amino acids (Σ EAAs) and sum of the branch-chain amino acids (Σ BCAAs; leucine + isoleucine + valine). **(B)** SDS-PAGE protein profiles of the aqueous extracts recovered from camelina seeds, analyzed in reducing conditions (RC, +BME) and non-reducing conditions (NRC, -BME). Right: schematic representation of the cruciferins and napins in the protein extracts recovered by aqueous extraction from camelina seeds. Abbreviation: MW = molecular weight.

Fig. 2 presents the AA compositions of the camelina seeds and of the defatted protein extracts recovered from the seeds. The AA compositions of eggs and bovine milk are also presented for comparison.

The comparative analysis of the AA compositions of the whole camelina seeds and the defatted protein aqueous extracts did not reveal any significant (*P* < 0.05) difference (Fig. 2). We therefore concluded that the multi-step refinery process applied to the camelina seeds that included aqueous extraction in water at pH 8 (Fig. 1) did not induce a fractionation of camelina seed proteins.

The camelina seed proteins analyzed in this study contained the nine EAAs that cannot be synthesized *de novo* by the human organism and must thus be supplied through diet: phenylalanine, valine, threonine, tryptophan, methionine, leucine, isoleucine, lysine and histidine. The amount of these EAAs in the camelina seed protein aqueous extracts was 36.1 ± 1.4 g/100 g proteins (Fig. 2). This is in agreement with values reported for defatted camelina meal (Li et al., 2014). The concentration of lysine was 4.8 ± 0.4 g/100 g proteins, in agreement with values reported for defatted camelina meal (Li et al., 2014). The three branched-chain amino acids (BCAAs), leucine, isoleucine and valine, accounted for 16.9 g/100 g proteins (Fig. 2). Camelina seed proteins also contained the non-essential amino acids (NEAAs), with the predominant glutamic acid, in agreement with the literature (Li et al., 2014; Zubr, 2003). The basic function of dietary proteins in human nutrition is to supply adequate amounts of EAAs to meet the metabolic needs, and an optimal dietary AA intake is necessary for the growth of body proteins. On the basis of their AA composition (Fig. 2A), camelina seed proteins recovered in the aqueous extracts could be considered as a good source of plant proteins with high nutritional potential, as already highlighted for camelina seeds and meals (Li et al., 2014; Zubr, 2003).

The AA compositions of camelina seeds and defatted protein aqueous extracts were compared with AA compositions of eggs (data calculated from (Dajnowska et al., 2023)) and bovine milk (data from (Claeys et al., 2014)) (Fig. 2). Eggs offer a complete profile of EAAs and are therefore recognized as a source of high-quality proteins (Drewnowski, 2010). Considered to be the perfect protein source, egg proteins serve as the standard for comparison with other protein sources (Dubin et al., 1994; Puglisi and Fernandez, 2022). Bovine milk proteins are also considered to be high-quality and complete proteins because they contain the nine EAAs in proportions resembling the AA requirements for human nutrition (Brestenský et al., 2018). Milk is especially rich in EAAs and BCAAs. Camelina seed proteins contained a lower amount of EAAs than eggs and milk (35 vs. 45 and 42 g/100 g proteins, respectively) (Fig. 2A). In particular, lysine and leucine were lower in camelina seeds and protein extracts than in eggs and milk (lysine: 4.8 vs. 7.7 and 8.2 g/100 g proteins, respectively; leucine: 7.1 vs. 9.2 and 8.8 g/100 g proteins, respectively) (Fig. 2A). The methionine content was also 2.8-fold lower in the camelina seed proteins extracted from the seeds and recovered in the protein aqueous extracts than in eggs (i.e., 1.61–1.64 and 4.61 g/100 g proteins, respectively). Camelina seed proteins could be used as an alternative plant-based protein source with an AA composition close to that of animal proteins.

Fig. 2B shows the SDS-PAGE protein profiles of the camelina seed aqueous extracts. The camelina seed protein profile determined by SDS-PAGE in non-reducing conditions showed that the aqueous extract contained a variety of seed storage proteins, mainly legumine-type globulins (11S) cruciferins (MW: 50 kDa; hexamer MW: 300 kDa) and napin-type albumins (2S) (MW: 15 kDa) (Fig. 2B). This is in agreement with the literature since these two types of seed storage proteins are abundant (about 80–85% of total seed proteins) in Brassicaceae oilseeds, including the crucifer camelina oilseeds (Wanasundara, 2011). Glutelin-type polypeptides (MW: approx. 15–20 kDa) were not observed. In non-reducing conditions, the higher density of the protein band related to the cruciferins was linked to their higher proportion compared to napins. This is in agreement with the literature since authors reported that 11S cruciferins and 2S napins account for about 60% and 20% of total proteins in mature Brassicaceae seeds, respectively (Boyle et al., 2018; Wanasundara, 2011). In reducing conditions, cruciferin and napin sub-units were characterized. Each polypeptide of cruciferin consists of a heavy α-chain (acidic, MW: approx. 30 kDa) and a light β-chain (basic, MW: approx. 20 kDa) linked with one disulfide bond (Wanasundara, 2011). Napin polypeptide consists of two basic sub-units, a short-chain sub-unit (MW: approx. 4.5 kDa) and a long-chain sub-unit (MW: approx. 10 kDa) after disruption of the 1:1 disulfide-linked complex (Wanasundara, 2011).

The nutritional quality of dietary proteins depends on the AA composition and protein profile, as well as on the digestibility (i.e., accessibility to digestive enzymes) and bioavailability. The protein quality of foods is characterized by the content of digestible AA in relation to their requirements for the human body and is expressed by the Digestible Indispensable Amino Acid Score (DIAAS) (Brestenský et al., 2018). Further experiments are then required to determine the DIAAS coefficient for camelina seed proteins. The 2S albumin napins found in Brassicaceae seeds are one of the three major groups of allergenic proteins of the prolamin superfamily (e.g., napins of mustard) (Wanasundara, 2011). The allergenicity of camelina seed proteins should also be investigated with a comparison between aqueous extraction from the seeds (Fig. 1) or extraction from the meal.

### 3.2 Camelina seed proteins recovered by aqueous extraction: electrostatic surface charge, solubility profile and physical stability determined as a function of pH

**Fig. 3:**
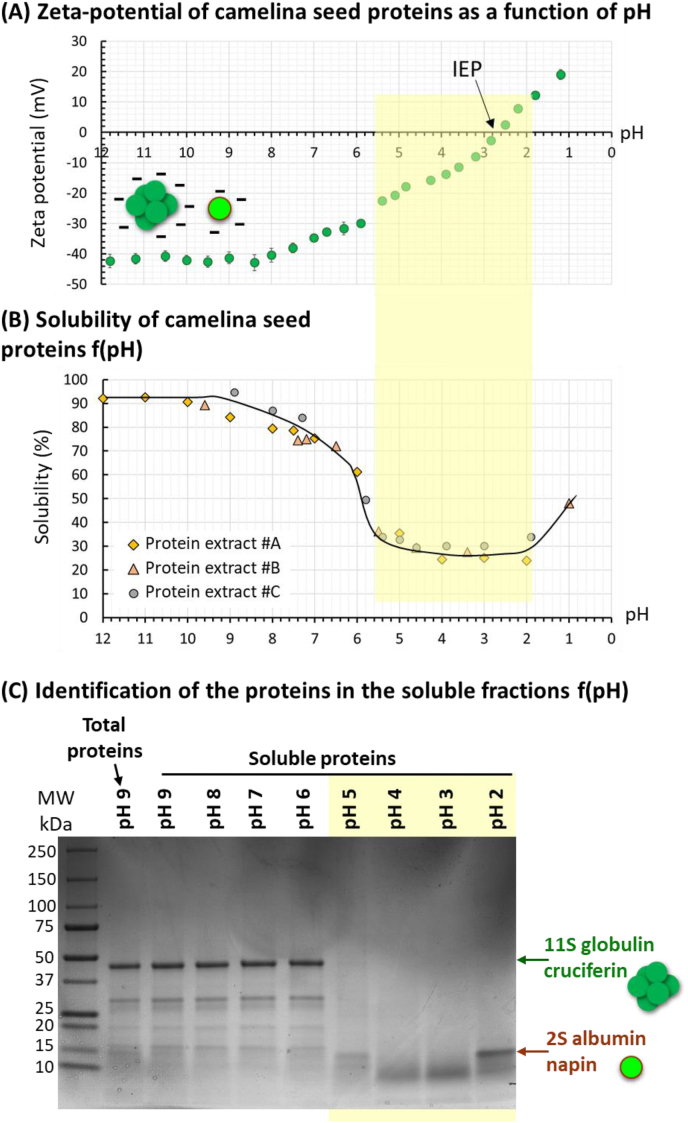
Camelina seed protein aqueous extracts: **(A)** zeta potential as a function of pH. A schematic representation of cruciferin (left) and napin (right) with negative charges at basic pH is proposed; **(B)** solubility profile of the proteins in water as a function of pH determined with three independent protein extracts (noted #A, #B, #C); and **(C)** SDS-PAGE protein profiles of the soluble fractions of camelina seed protein aqueous extracts recovered as a function of pH (SDS-PAGE in non-reducing conditions; -BME). Abbreviations: IEP = isoelectric point; MW = molecular weight.

The electrostatic surface charge of the proteins is an important characteristic that determines their functional properties in bulk and in oil-in-water emulsions. The electrostatic surface charge of the camelina seed proteins was therefore determined by ζ-potential analysis as a function of pH (Fig. 3A).

The ζ-potential of the proteins evolved from negative to positive with decreasing pH. In the pH range from 12 to 8.4, the ζ-potential was −41.9 ± 0.8 mV. The absolute values of ζ-potential decreased with pH and reached the isoelectric point (IEP; ζ = 0 mV) at pH 2.6. For pHs below the IEP, the ζ-potential values were positive, with ζ-potential = +19.0 ± 1.6 mV at pH 1.2, indicating a net positive charge of the camelina seed proteins.

The solubility of proteins is a key parameter involved in their functional properties such as the stabilization of oil droplets in oil-in-water emulsions and the overall physical stability of the emulsions. The solubility profile of the camelina seed proteins contained in the defatted aqueous extracts was therefore investigated as a function of pH (i.e., an extrinsic factor). In this study, the solubility of camelina seed proteins recovered by aqueous extraction was defined as the amount of soluble proteins in the supernatant of a dispersion after centrifugation under defined conditions. Fig. 3B presents the solubility profile of the camelina seed proteins at different pH values. Notably, the solubility of the camelina seed proteins exhibited pH dependence. Changes in the solubility of the camelina seed proteins as a function of pH showed the highest solubility values at alkaline pH (pH 7–12; more than 75% solubility), and the lowest solubility values between pH 5 and 2. An increase in the solubility of the proteins in extreme acidic conditions below pH 2 was observed. The changes in the solubility of camelina seed proteins as a function of pH corresponded to the characteristic U-shape curve of the protein solubility (Ladjal-Ettoumi et al., 2016). Protein solubility varied with the electrostatic surface charge of the proteins (Fig. 3A). The lowest solubility values corresponded to pH values close to the IEP of the camelina seed proteins determined with the same aqueous extracts (Fig. 3A). The protein profiles of the soluble protein fractions determined by SDS-PAGE in non-reducing conditions revealed compositional changes as a function of pH (Fig. 3C). For pH values above pH 6, the composition of the soluble fraction was similar to the total proteins with 11S globulin cruciferins (MW: 48 kDa) and 2S water-soluble albumin napins (MW: 15 kDa). As of pH 5 and at acidic pH values, only the 2S albumin proteins (napins) were present in the soluble protein fraction. This was interpreted as the insolubility of the 11S globulin cruciferins below pH 6 and their sedimentation. This is in agreement with the ionization of Brassicaceae seed storage napins at acidic pHs and the solubility of this albumin fraction reported for pH 4–3 (Wanasundara, 2011).

**Fig. 4:**
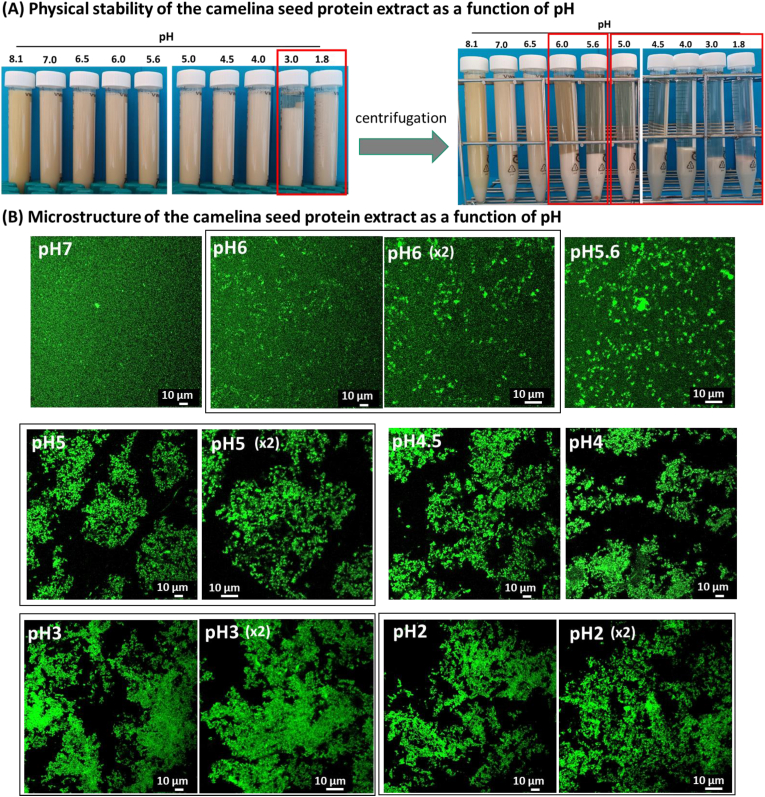
Physical stability of the defatted camelina seed protein aqueous extract as a function of pH: **(A)** photo of the tubes showing the physical stability of the sample as a function of pH; **(B)** confocal laser scanning microscopy (CLSM) images showing the microstructure of the defatted camelina seed protein aqueous extract as a function of pH; the proteins were stained with the fast green FCF fluorescent dye (green color). The scale bars are indicated in the figures. (For interpretation of the references to color in this figure legend, the reader is referred to the Web version of this article.)

The physical stability of the camelina seed protein extracts was investigated as a function of pH (Fig. 4).

After adjustment of the pH of the protein extract, the samples spontaneously sedimented for acidic pH values, i.e., pH 3 and pH 1.8. This pH range is close to the IEP of the camelina seed proteins for which the net charge of the proteins is zero, which minimizes electrostatic repulsion, leading to protein aggregation and precipitation (Grossmann and McClements, 2023). After centrifugation performed to accelerate the mechanism of phase separation, the samples sedimented for pH 6 and below. In the photo of the tubes (Fig. 4A), the sample adjusted at pH 8.1 exhibited a brown color due to the presence of phenolic compounds and, more particularly, to rutin (Lopez et al., 2023b). Above pH 4.5, the supernatant of the samples was colored (Fig. 4A), which was interpreted as the presence of soluble proteins and phenolic compounds. CLSM images showed, at the microscopic scale, that for pH 7 the camelina seed proteins were homogeneously distributed in the sample and then soluble. For pH 6, proteins started to form small aggregates at the same time as a continuous phase of soluble proteins. On the basis of the protein solubility and identification of the soluble proteins (Fig. 3), we deduced that these protein aggregates corresponded to cruciferin particles that were insoluble and sedimented. As a function of the decrease in pH, the size of the protein aggregates increased by the accumulation of insoluble proteins. The formation of large aggregates of insoluble proteins observed at the microscopic scale was responsible for the sedimentation of camelina seed proteins below pH 6 observed at the macroscopic level. The reduction of the negative charge of the proteins as a function of the decrease in pH decreased the electrostatic repulsion and was responsible for protein aggregation and precipitation (Grossmann and McClements, 2023). The aggregation of proteins below pH 6 resulted in their reduced solubility (Fig. 3B). The sedimentation of the camelina seed proteins below pH 6 was related to their high density compared to the density of water.

The main proteins, cruciferins and napins of camelina seed exhibit different solubilities as a function of pH that lead to the aggregation and precipitation of cruciferin at acidic pHs. They could be fractionated by acid precipitation of cruciferin below pH 6 and the napins could be recovered in the soluble fraction, as already performed for other Brassicaceae proteins (Ntone et al., 2021).

### 3.3 Protein-coated oil droplets: microstructure and physical stability of the emulsions as a function of pH

The defatted camelina seed protein extracts (protein content: 10.0 ± 0.8 mg/mL) were used in combination with a commercial camelina seed oil to prepare O/W emulsions (3 %wt oil). The O/W emulsions were prepared at pH 8 in order to benefit from the high solubility of both the cruciferins and napins dispersed in the defatted camelina seed aqueous extracts (Fig. 3B) and to optimize their emulsifying properties. O/W emulsions characterized by various size distributions of the oil droplets were prepared by varying the homogenization pressure applied. The microstructure and surface properties of the oil droplets were characterized and the physical stability of the O/W emulsions was studied as a function of pH.

**Fig. 5:**
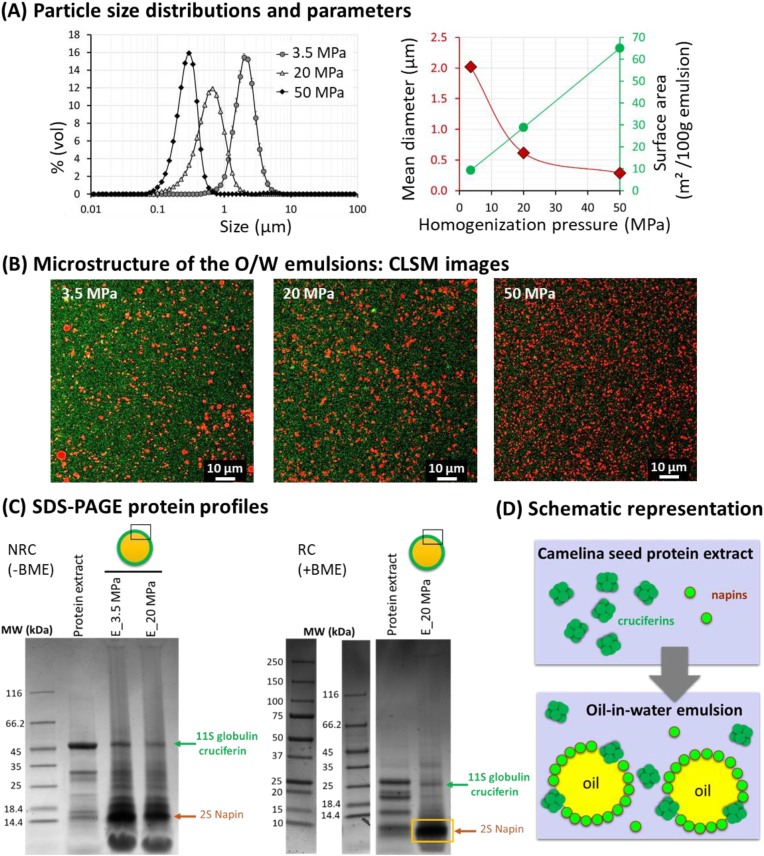
Oil-in-water emulsions (3 %wt oil) prepared with defatted camelina seed protein aqueous extracts. **(A)** Oil droplet size distributions and parameters: (left) size distributions of the oil droplets stabilized with the camelina seed protein extract at pH 8.0 prepared at various homogenization pressures as indicated in the figure; (right) changes in the mean diameter and surface area as a function of the homogenization pressure applied. **(B)** Confocal laser scanning microscopy (CLSM) images of the emulsions; the emulsions were stained with fast green FCF for the proteins and Nile red for the oil. **(C)** SDS-PAGE protein profiles of camelina seed protein extract and oil droplets in the emulsions (denoted E_). **(D)** Schematic representation of the camelina seed proteins in the aqueous extract and the preferential localization of napins at the oil/water interface in the emulsions. Abbreviations: MW = protein molecular weight marker; NRC = non-reducing conditions (-BME); RC = reducing conditions (+BME). (For interpretation of the references to color in this figure legend, the reader is referred to the Web version of this article.)

The size distributions of the oil droplets in the O/W emulsions are presented in Fig. 5A.

The comparative analysis of particle size measurements performed without and in the presence of SDS (i.e., to disrupt oil droplet flocculation) revealed the absence of aggregation of oil droplets in the emulsions at pH 8. The increase in the homogenization pressure from 3.5 MPa to 50 MPa induced a decrease in the mean diameter of the lipid droplets from 2.02 ± 0.07 to 0.29 ± 0.02 μm, and an increase in the surface area from 9.3 to 65.1 m^2^ per 100 g of emulsion. The effective emulsification required camelina seed proteins to migrate to the oil/water interface and form a film around each oil droplet. These results showed that the amount of proteins present in the camelina seed aqueous extract (10.0 ± 0.8 mg/mL) was sufficient to cover the surface created by 3 %wt of camelina oil upon homogenization. The ζ-potential values of the emulsions were similar regardless of the size of the oil droplets: 31.2 ± 1.4 mV at pH 8. The negative values of the ζ-potential contributed to the electrostatic repulsion between the oil droplets and to the physical stability of the emulsions. CLSM images of the emulsions are presented in Fig. 5B. The increase in the homogenization pressure induced the fractionation of the oil into a higher number of oil droplets of 0.29 μm (P = 50 MPa) than oil droplets of 2.02 μm (P = 3.5 MPa).

In this study, O/W emulsions were efficiently prepared with protein extracts recovered from camelina seeds by aqueous processing. These protein extracts exhibited a natural chemical complexity typically containing multiple species of proteins, and were mainly composed of camelina seed cruciferins and napins (Fig. 5C). Interestingly, the profiles of the proteins adsorbed at the surface of the oil droplets and of the protein extract used to prepare the O/W emulsions were different. The SDS-PAGE protein profile of the oil/water interface revealed the co-adsorption of cruciferins and napins at the oil/water interface, but with a higher proportion of napins than cruciferins (Fig. 5C). The difference determined between the relative proportion of cruciferins and napins in the aqueous dispersion and at the surface of the oil droplets was interpreted as a preferential adsorption of napins at the oil/water interface. Fig. 5D shows a schematic representation of camelina seed proteins in the aqueous extract and adsorbed at the surface of the oil droplets. Our results are different from previous results reported for rapeseed proteins. Indeed, with a natural mixture of rapeseed proteins containing both napins and cruciferins, authors reported that only napins were present at the surface of the oil droplets (Ntone et al., 2021). The mechanisms leading to adsorption of proteins at oil/water interfaces are mainly driven by hydrophobic interactions. At the oil/water interface, the proteins are denatured to adopt a stable conformation and to minimize the interfacial free energy. The dominance of napins at the surface of the oil droplets was ascribed to their small size and their unique Janus-like structure since 45% of the amino acids are hydrophobic and primarily located on one side of the protein, as previously reported for rapeseed napins (Ntone et al., 2021). Furthermore, authors reported that albumins such as napins are highly surface-active in stabilizing O/W interfaces compared to globulins such as cruciferins (Wanasundara, 2011). The globulin cruciferins (11S proteins) have been reported to exhibit low emulsifying ability because of their globular conformation that is maintained at the O/W interface and contributes to the low surface activity (Wanasundara, 2011). Cruciferins, which are hexamers with a MW of 300 kDa, exhibit a larger size than napins and a more homogeneous distribution of the hydrophobic domains, which is not favorable for their adsorption at the O/W interface, as previously discussed for rapeseed cruciferins (Ntone et al., 2021). Some authors have reported that napins alone (i.e., in the absence of cruciferins) were more efficient emulsifiers than cruciferins alone to stabilize O/W emulsions (Krause and Schwenke, 2001). However, few studies have investigated the competition between napins and cruciferins from a natural protein extract such as camelina seed protein aqueous extract.

The physical stability of the O/W emulsions was investigated as a function of time in storage at 4 °C (results not shown). The emulsion prepared at 3.5 MPa and containing the large oil droplets (i.e., 2 μm) showed creaming as a function of time. The other emulsions containing small oil droplets (i.e., <1 μm) did not show any creaming upon storage. The creaming was therefore attributed to the large size of the oil droplets. The emulsions did not exhibit coalescence upon storage at 4 °C for 1 month. These results highlighted the high capacity of camelina seed proteins recovered by aqueous extraction to stabilize the oil droplets in O/W emulsions. Camelina seed proteins are therefore good plant-based emulsifiers that could be used as alternatives, e.g., to dairy proteins, for the stabilization of food emulsions.

**Fig. 6:**
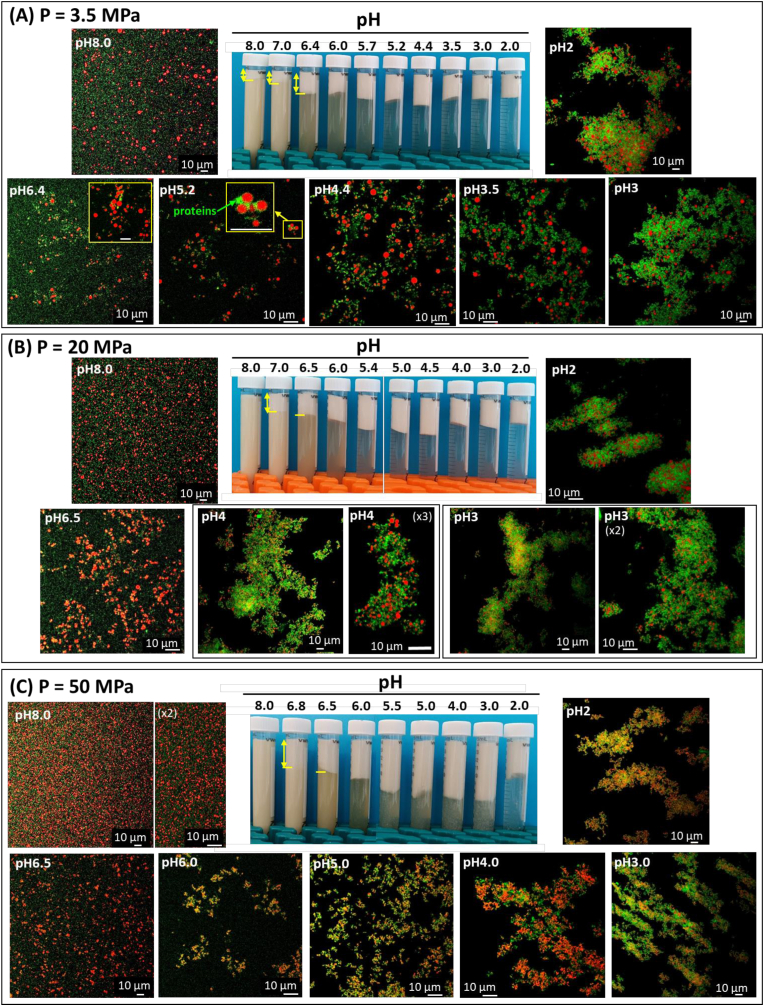
Physical stability as a function of pH of the oil-in-water emulsions prepared with camelina seed protein aqueous extracts and homogenized at various pressures, referred to as P: **(A)** P = 3.5 MPa, **(B)** P = 20 MPa and **(C)** P = 50 MPa. For each emulsion, the photo of the tubes shows the physical stability of the emulsion as a function of pH and the confocal laser scanning microscopy (CLSM) images show the microstructure as a function of pH. In the CLSM images, the proteins were stained with the fast green FCF fluorescent dye (green color) and the oil droplets were stained with Nile red (red color). (For interpretation of the references to color in this figure legend, the reader is referred to the Web version of this article.)

The physical stability of the O/W emulsions containing camelina seed protein-coated oil droplets of various sizes was examined as a function of pH (Fig. 6).

**Fig. 7:**
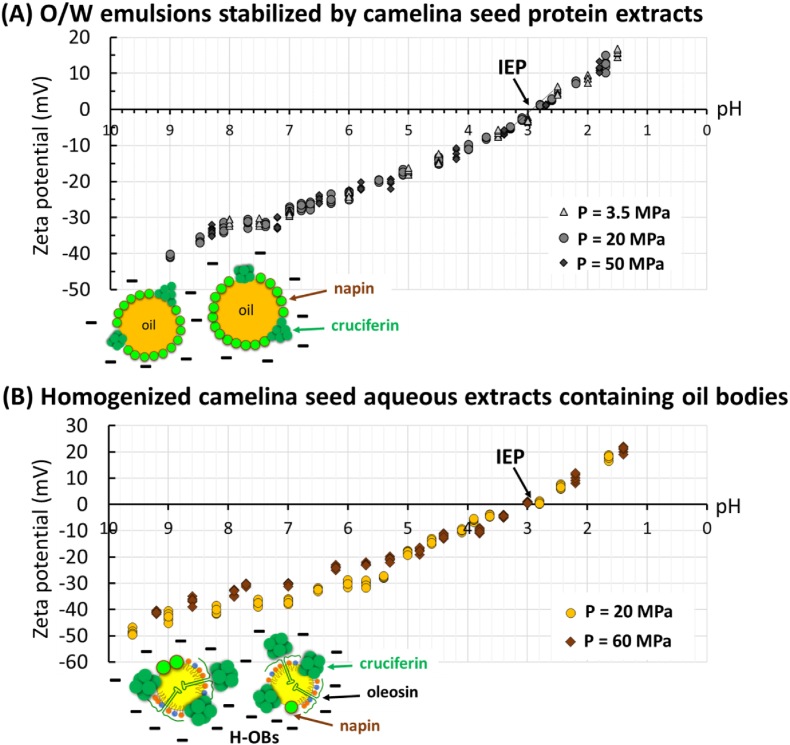
Zeta potential as a function of pH determined for **(A)** oil-in-water emulsions stabilized by camelina seed protein aqueous extracts and homogenized at various pressures P as indicated in the figure; and **(B)** camelina seed whole aqueous extracts containing natural oil bodies homogenized at P = 20 MPa and P = 60 MPa leading to the formation of homogenized oil bodies (H-OBs).

At pH 8, an oil droplet-rich phase was formed at the top of the tube at a homogenization pressure of 3.5 MPa but not at homogenization pressures of 20 and 50 MPa. This was interpreted as a result of the large size of the oil droplets (2.02 μm). At pH 7, aggregated oil droplets were recovered at the top of the tubes regardless of the size of the oil droplets. Particle size measurements confirmed the aggregation of oil droplets. We deduced that the decrease in pH from 8 to 7 affected the electrostatic surface charge of the proteins (decrease below −30 mV; Fig. 7A) and induced aggregation of oil droplets that moved upward. This is in agreement with the literature since in emulsions prepared at pH 7 and stabilized by rapeseed proteins including cruciferins and napins, authors reported the aggregation of oil droplets (Ntone et al., 2021). These authors reported that cruciferins connected to the napins could contribute to the formation of the lipid droplet aggregates that they observed in the emulsion at pH 7 (Ntone et al., 2021). In all the emulsions, the decrease in the pH induced phase separation with a concentration of the sample at the top of the tubes. CLSM images showed the formation of oil droplet aggregates connected by camelina seed proteins. The high fluorescence intensity around the oil droplets (Fig. 6A, pH 5.2, insert) was interpreted as an increase in the amount of proteins adsorbed at the surface of the oil droplets or to changes in the organization of proteins due to their aggregation. On the basis of the protein solubility measurements associated with SDS-PAGE protein profiles (Fig. 3B and C), we deduced that the 11S globulin cruciferins were involved in the aggregation of the oil droplets. An increase in the size of the oil droplets by coalescence was not observed regardless of the pH, highlighting the physical stability of the interfacial films surrounding each oil droplet. The presence of aggregates of oil droplets and proteins at the top of the tubes was governed by the lower density of oil than water.

The ζ-potential of the protein-coated oil droplets of various sizes was determined as a function of pH and is presented in Fig. 7A. The ζ-potential of the protein-coated oil droplets evolved from negative to positive with decreasing pH, without any significant difference between the emulsions prepared with different homogenization pressures. The IEP (ζ = 0 mV) corresponded to pH 2.8–3. The trends in ζ-potential values measured in this study as a function of pH for camelina seed proteins are in agreement with the literature for Brassicaceae proteins such as those from rapeseeds (Ntone et al., 2021). The IEP measured in this study for camelina seed protein-coated oil droplets was lower than the IEP reported for oil droplets covered by Brassicaceae proteins from rapeseeds (Ntone et al., 2021).

### 3.4 Homogenized camelina seed OBs: microstructure, surface properties and physical stability of the aqueous extracts as a function of pH

In previous works, we showed that the high-pressure homogenization of whole aqueous extracts recovered from oilseeds (e.g., hemp seeds (Lopez et al., 2021)) and nuts (e.g., walnuts (Lopez et al., 2023a)) leads to the adsorption of exogenous proteins at the surface of the oil bodies (OBs). In this part of the study, we aimed at determining the impact of camelina seed proteins adsorbed at the surface of homogenized camelina seed OBs on the physical stability of the emulsions as a function of pH.

#### 3.4.1 Impact of the homogenization on the microstructure and surface composition of camelina seed OBs

The whole aqueous extracts recovered from camelina seeds contained both camelina seed proteins and oil bodies (OBs), as already reported (Lopez et al., 2023b). The proteins could play a role in the surface composition and properties of camelina seed OBs modified under shear by high-pressure homogenization. This has already been reported for homogenized hemp seed OBs and homogenized walnut OBs (Lopez et al., 2021, 2023a). The microstructure of homogenized camelina seed aqueous extracts, and the size distributions and zeta potential of homogenized camelina seed OBs were therefore investigated.

**Fig. 8:**
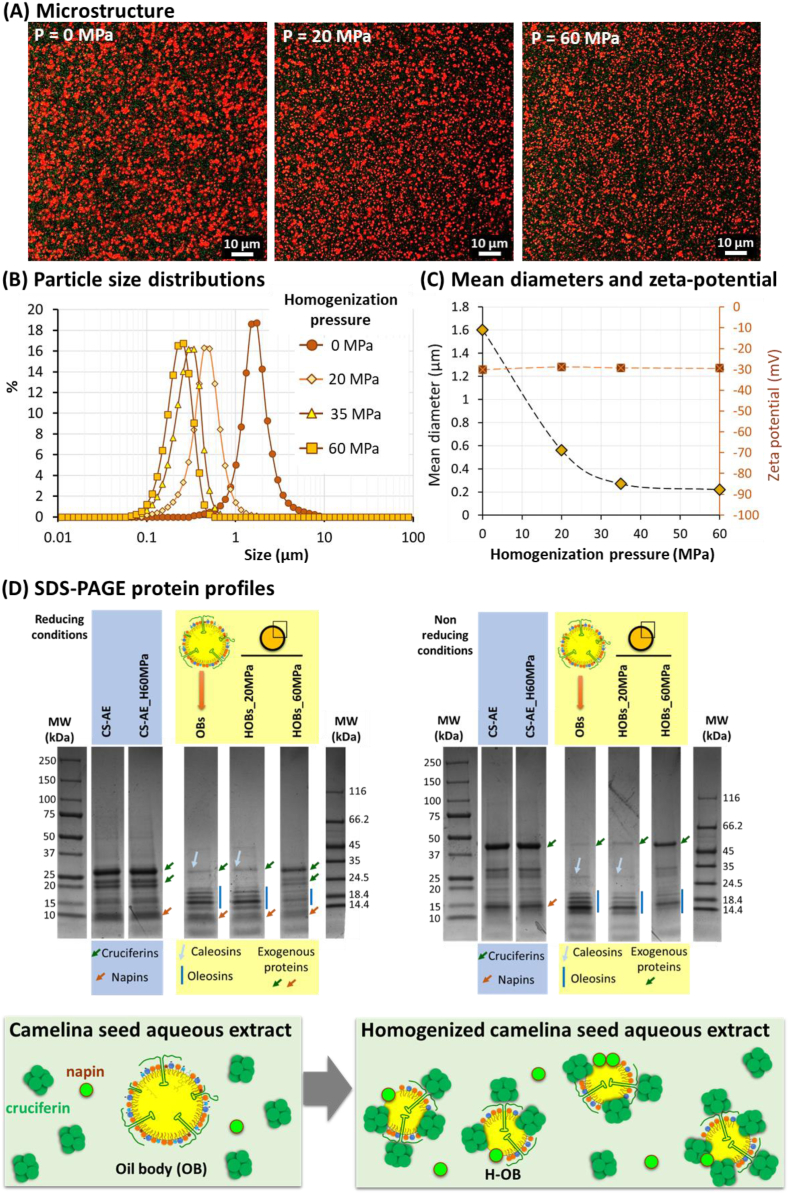
Impact of homogenization on the microstructure and surface composition of oil bodies in camelina seed whole aqueous extracts: **(A)** confocal laser scanning microscopy (CLSM) images of the camelina seed whole aqueous extract containing natural oil bodies (P = 0 MPa) and after homogenization at P = 20 MPa and P = 60 MPa. In the CLSM images, the proteins were stained with the fast green FCF fluorescent dye (green color) and the oil droplets were stained with Nile red (red color); **(B)** oil droplet size distributions determined after homogenization of the camelina seed whole aqueous extract at different pressures as indicated in the figure; **(C)** mean diameters of the oil bodies and zeta potential as a function of the homogenization pressure; **(D)** SDS-PAGE protein profiles and schematic representation of the impact of homogenization on the surface composition of camelina seed oil bodies. Abbreviations: P = homogenization pressure; MW = molecular weight; CS-AE = camelina seed aqueous extract; CS-AE_H60MPa = camelina seed aqueous extract homogenized at 60 MPa; OBs = natural oil bodies; HOBs_20MPa and HOBs_60MPa = homogenized oil bodies recovered after homogenization at 20 MPa and 60 MPa, respectively. (For interpretation of the references to color in this figure legend, the reader is referred to the Web version of this article.)

Whole camelina seed aqueous extracts (lipid content: 3.0 ± 0.3 g/100 g, protein content: 10.0 ± 0.8 mg/mL) were homogenized at 3.5, 20, 35 and 60 MPa. The increase in the homogenization pressure induced a decrease in the size of homogenized camelina seed OBs (H-OBs) compared to natural OBs, as observed in the CLSM images (Fig. 8A). The resolution of CLSM did not allow the observation of the camelina seed proteins because of their small size and absence of protein bodies, contrary to the aqueous extracts recovered from hemp seeds, chia seeds and walnuts (Lopez et al., 2021, 2023b, 2023a). The size distributions of natural camelina seed OBs and H-OBs were determined (Fig. 8B). The mean diameters were calculated (Fig. 8C). Homogenizing the camelina seed whole aqueous extract at 20 MPa decreased the mean diameter from 1.62 ± 0.2 μm to 0.56 ± 0.15 μm. The whole aqueous extracts homogenized at 35 MPa and 60 MPa contained H-OBs with mean diameters of 0.27 ± 0.01 μm and 0.22 ± 0.01 μm, respectively. The decrease in the mean diameter of the camelina seed H-OBs as a function of the increase in the homogenization pressure showed: (i) that the pressure applied was efficient; and (ii) that the protein content in the aqueous extract was not a limiting parameter. Indeed, the protein content permitted the stabilization of the surface of H-OBs created under pressure in the homogenizer. Upon homogenization, the camelina seed proteins present in the aqueous extract migrated to the O/W interface. The proteins located at the O/W interface were emulsifying agents that formed a macromolecular layer around the H-OBs.

The ζ-potential values of natural OBs and H-OBs were similar: ζ_0MPa_ = −30.1 ± 1.1 mV, ζ_60MPa_ = −29.5 ± 1.2 mV (Fig. 7B). The negative ζ-potential values at pH 8 were involved in electrostatic repulsions between the H-OBs. The surface properties of H-OBs prevented their aggregation and contributed to their homogeneous dispersion in the volume of the product, as observed in the CLSM images (Fig. 8A).

SDS-PAGE under reducing and non-reducing conditions permitted the identification of the protein profiles in the camelina seed aqueous extracts and after homogenization at 20 MPa, and the determination of the proteins adsorbed at the surface of natural OBs and H-OBs (Fig. 8D). In the aqueous extracts, the main proteins were cruciferins (MW of 20–30 kDa in reducing conditions; MW of 48 kDa in non-reducing conditions) and napins (MW of 10 kDa in reducing conditions; MW of 15 kDa in non-reducing conditions). The high-pressure homogenization of the whole protein aqueous extract did not alter the protein profile. At the surface of camelina seed natural OBs, the oleosins (MW: 15–23 kDa) and caleosins (MW: 30 kDa) were identified, in agreement with the literature (Nikiforidis et al., 2014; Tzen et al., 1993). The oleosins are major structural proteins of Brassicaceae oilseeds. The presence of non-OB exogenous proteins from the camelina seed aqueous extract, i.e., cruciferins and napins, was also identified at the surface of camelina seed OBs. The higher intensity of the band related to napins (MW 10 kDa) compared to cruciferins (MW 30 kDa) was interpreted as a higher proportion of napins than cruciferins at the surface of OBs (Fig. 8D, left, reducing conditions).

Homogenization of the camelina seed whole aqueous extracts induced a reduction of the size of OBs (Fig. 8A, B, C). Homogenization also induced the adsorption of non-OB proteins at the surface of the H-OBs, mainly the cruciferins and napins (Fig. 8D). In the whole aqueous extract homogenized at 60 MPa, the high intensity of the bands corresponding to cruciferin (30 + 20 kDa in reducing conditions; 48 kDa in non-reducing condition) was interpreted as a greater amount of cruciferins than napins at the surface of the H-OBs. The decrease in the relative proportion of oleosins and caleosins at the surface of the H-OBs as a function of the increase in the homogenization pressure was deduced from the decrease in the relative density of the bands corresponding to these specialized integral membrane proteins. This was combined with the presence of non-OB proteins from the aqueous phase, mainly the cruciferins. The high cohesivity of the camelina seed OBs could explain the presence of oleosins and probably caleosins at the surface of camelina seed H-OBs homogenized at 60 MPa (mean diameter: 0.22 ± 0.01 μm). This was already discussed for walnut H-OBs (Lopez et al., 2023a) and hemp seed H-OBs (Lopez et al., 2021). The secondary structures of oleosins allow them to remain stable in the phospholipid monolayer on the surface of the OBs (Jolivet et al., 2004). Gray et al. (2010) reported that oleosins act as a barrier to oxidizing agents such as hydroperoxides and oxygen. Interestingly, the oleosins present at the surface of camelina seed H-OBs could play a key role, together with other antioxidants (tocopherols), in the protection of PUFAs against oxidation.

These experiments revealed that the interfacial layer of camelina seed H-OBs is composed of both specific membrane proteins from natural OBs (i.e., oleosins, caleosins) and non-OB exogenous proteins such as cruciferins and napins with a high proportion of cruciferins (Fig. 8D). Interestingly, the relative proportion of cruciferins and napins at the surface of H-OBs was different from their proportion at the surface of the emulsions prepared from oil and the camelina seed protein aqueous extract devoid of OBs where the napins were the most abundant proteins (Fig. 5C). We hypothesized that cruciferins could interact with oleosins and polar lipids at the surface of H-OBs and that the polar lipids surrounding OBs could limit the adsorption of napins. A schematic representation is proposed (Fig. 8D, bottom).

#### 3.4.2 Physical stability of homogenized camelina seed whole aqueous extracts as a function of pH

**Fig. 9:**
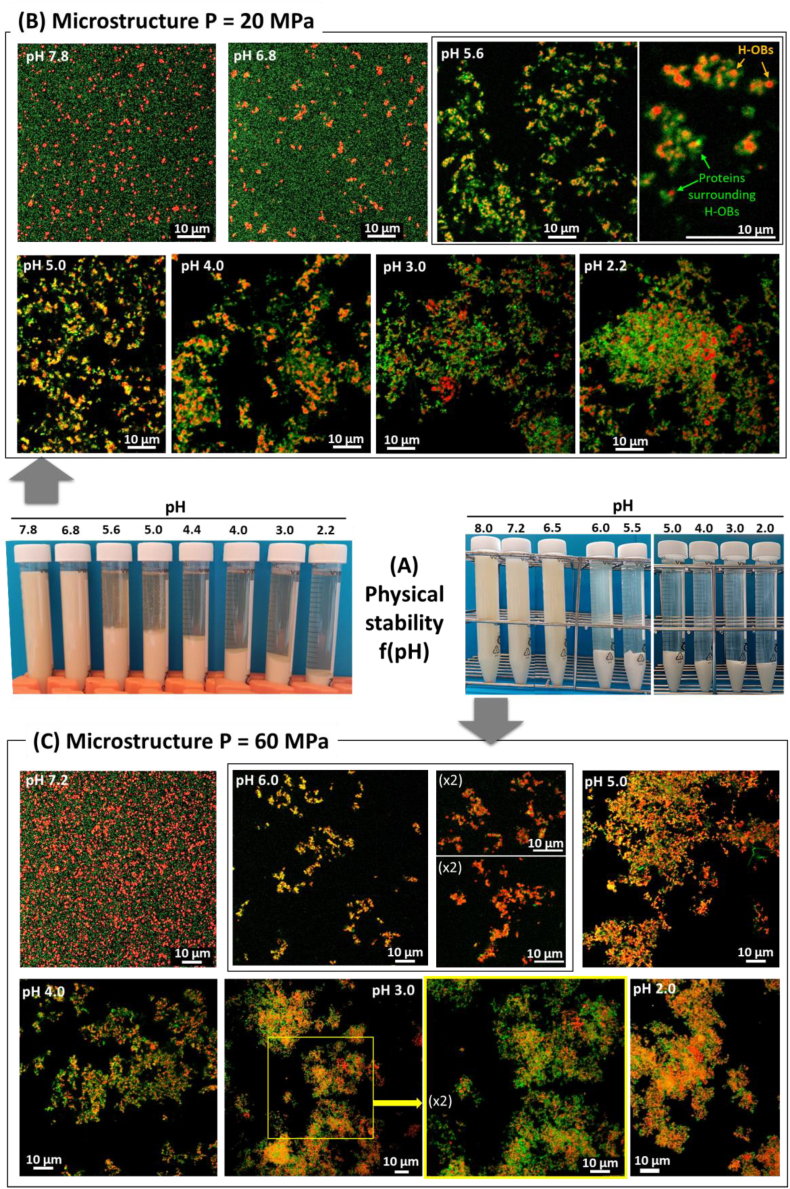
Physical stability of the homogenized camelina seed whole aqueous extracts as a function of pH: **(A)** Photos of the tubes as a function of pH; left: camelina seed whole aqueous extract homogenized at P = 20 MPa; right camelina seed whole aqueous extract homogenized at P = 60 MPa; **(B)** confocal laser scanning microscopy (CLSM) images showing the microstructure of the camelina seed aqueous extract homogenized at P = 20 MPa; **(C)** CLSM images showing the microstructure of the camelina seed aqueous extract homogenized at P = 60 MPa. In the CLSM images, the proteins were stained with the fast green FCF fluorescent dye (green color) and the oil droplets were stained with Nile red (red color). (For interpretation of the references to color in this figure legend, the reader is referred to the Web version of this article.)

The physical stability of the aqueous extracts homogenized at 20 MPa and 60 MPa was investigated as a function of pH (Fig. 9). Below pH 6.8–6.5, the homogenized aqueous extracts showed a phase separation with sedimentation. CLSM images revealed the formation of aggregates in which the H-OBs were entrapped in the protein network. We deduced that the camelina seed H-OBs covered by proteins, i.e., specific OB proteins and non-OB proteins such as cruciferins and napins, act as a nucleus on which the other proteins dispersed in the surrounding aqueous phase tend to aggregate as a function of the decrease in pH. Surprisingly, the decrease in pH induced sedimentation of the complexes formed by the H-OBs and the proteins. Indeed, in the oil-in-water emulsions prepared from oil and the protein aqueous extracts (Fig. 6), the aggregated oil droplets moved to the top of the tubes after their destabilization by the pH. In the case of H-OBs, the sedimentation of the aggregates showed that the proteins surrounding the H-OBs governed the density of the aggregates, which was higher than the density of water. The high proportion of cruciferins at the surface of the H-OBs (Fig. 8D) and the decrease in the solubility of cruciferins below pH 6 (Fig. 2) could explain this behavior.

## 4 Conclusion

Exploring the biodiversity of plant-based proteins produced by sustainable crops is of primary importance in the current context of food protein transition. The green multi-step aqueous process applied to camelina seeds successfully allowed the recovery of protein extracts. This is a technological innovation compared to the classical process of seed pressing and extraction of the proteins from the meal. The aqueous extracts contained proteins, mainly the 11S globulin cruciferin and 2S albumin napin, which are composed of the nine essential amino acids. The solubility of the aqueous protein extracts evolved as a function of pH according to the characteristic U-shape, with minimal values between pH 5 and 2. The exploration of the capacity of camelina seed proteins to stabilize oil droplets in oil-in-water emulsion prepared from oil and defatted protein extracts, and to stabilize oil bodies in homogenized camelina seed whole aqueous extracts revealed different surface compositions of the oil droplets. In the emulsions, the napins were preferentially adsorbed at the oil/water interface. At the surface of homogenized oil bodies, oleosins were present together with a high amount of the exogenous protein cruciferins. The camelina seed protein aqueous extracts (protein content: 10 mg/mL) exhibited good emulsifying and stabilizing properties in the preparation of 3 %wt oil-in-water emulsions. The physical stability of the emulsions was governed by the solubility of camelina seed proteins as a function of pH: below pH 6, the aggregation of protein-coated oil droplets induced phase separation in the emulsions. These results revealed the interesting nutritional and functional properties of camelina seed proteins recovered by aqueous extraction as natural plant-based emulsifiers to stabilize oil-in-water emulsions for innovative and sustainable food applications. This study will therefore encourage the development of camelina seed crops in order to contribute to the food protein transition, and to reduce the carbon footprint and the environmental impact of foods.

## CRediT authorship contribution statement

**Christelle Lopez:** Conceptualization, Supervision, Investigation, Formal analysis, Validation, Writing – original draft, Writing – review & editing. **Hanitra Rabesona:** Conceptualization, Investigation, Formal analysis, Validation, Writing – original draft, Writing – review & editing. **Valérie Beaumal:** Investigation, Formal analysis, Validation, Writing – original draft, Writing – review & editing. **Hélène Sotin:** Investigation, Formal analysis, Validation, Writing – original draft, Writing – review & editing. **Bruno Novales:** Conceptualization, Writing – original draft, Writing – review & editing. **Marc Anton:** Conceptualization, Writing – original draft, Writing – review & editing.

## Declaration of competing interest

The authors declare that they have no known competing financial interests or personal relationships that could have appeared to influence the work reported in this paper.

## Data Availability

Data will be made available on request.

## References

[bib1] Abad A., Shahidi F. (2021). Fatty acid, triacylglycerol and minor component profiles affect oxidative stability of camelina and sophia seed oils. Food Biosci..

[bib2] Acevedo-Fani A., Dave A., Singh H. (2020). Nature-assembled structures for delivery of bioactive compounds and their potential in functional foods. Front. Chem..

[bib3] Arshad M., Mohanty A K., Acker R.V., Riddle R., Todd J., Khalil H., Misra M. (2022). Valorization of camelina oil to biobased materials and biofuels for new industrial uses: a review. RSC Adv..

[bib4] Berti M., Gesch R., Eynck C., Anderson J., Cermak S. (2016). Camelina uses, genetics, genomics, production, and management. Ind. Crop. Prod..

[bib5] Boyle C., Hansen L., Hinnenkamp C., Ismail B.P. (2018). Emerging camelina protein: extraction, modification, and structural/functional characterization. JAOCS (J. Am. Oil Chem. Soc.).

[bib6] Brestenský M., Nitrayová S., Patras P., Nitray J. (2018). Dietary requirements for proteins and amino acids in human nutrition. Curr. Nutr. Food Sci..

[bib7] Campos B.E., Dias Ruivo T., da Silva Scapim M.R., Madrona G.S., de C., Bergamasco R. (2016). Optimization of the mucilage extraction process from chia seeds and application in ice cream as a stabilizer and emulsifier. LWT - Food Sci. Technol. (Lebensmittel-Wissenschaft -Technol.).

[bib8] Claeys W.L., Verraes C., Cardoen S., De Block J., Huyghebaert A., Raes K., Dewettinck K., Herman L. (2014). Consumption of raw or heated milk from different species: an evaluation of the nutritional and potential health benefits. Food Control.

[bib9] Clavijo-Bernal E.J., Martínez-Force E., Garcés R., Salas J.J., Venegas-Calerón M. (2024). Biotechnological Camelina platform for green sustainable oleochemicals production. OCL.

[bib10] Dajnowska A., Tomaszewska E., Świątkiewicz S., Arczewska-Włosek A., Dobrowolski P., Domaradzki P., Rudyk H., Brezvyn O., Muzyka V., Kotsyumbas I., Arciszewski M.B., Muszyński S. (2023). Yolk fatty acid profile and amino acid composition in eggs from hens supplemented with ß-Hydroxy-ß-Methylbutyrate. Foods.

[bib11] Drewnowski A. (2010). The Nutrient Rich Foods Index helps to identify healthy, affordable foods 1234. Am. J. Clin. Nutr..

[bib12] Dubin S., McKee K., Battish S. (1994). Essential amino acid reference profile affects the evaluation of enteral feeding products. J. Am. Diet Assoc..

[bib13] Fabre J.-F., Lacroux E., Gravé G., Mouloungui Z. (2020). Extraction of camelina mucilage with ultrasound and high flow rate fluid circulation. Ind. Crop. Prod..

[bib14] Garcia F.L., Ma S., Dave A., Acevedo-Fani A. (2021). Structural and physicochemical characteristics of oil bodies from hemp seeds (Cannabis sativa L.). Foods.

[bib15] Ghidoli M., Pesenti M., Colombo F., Nocito F.F., Pilu R., Araniti F. (2023). Camelina sativa (L.) Crantz as a promising cover crop species with Allelopathic potential. Agron..

[bib16] Gray D.A., Payne G., McClements D.J., Decker E.A., Lad M. (2010). Oxidative stability of Echium plantagineum seed oil bodies. Eur. J. Lipid Sci. Technol..

[bib17] Grossmann L., McClements D.J. (2023). Current insights into protein solubility: a review of its importance for alternative proteins. Food Hydrocolloids.

[bib18] Jolivet P., Roux E., d'Andrea S., Davanture M., Negroni L., Zivy M., Chardot T. (2004). Protein composition of oil bodies in Arabidopsis thaliana ecotype WS. Plant Physiol. Biochem..

[bib19] Karvonen H.M., Aro A., Tapola N.S., Salminen I., Uusitupa M.I. j, Sarkkinen E.S. (2002). Effect of alpha-linolenic acid-rich Camelina sativa oil on serum fatty acid composition and serum lipids in hypercholesterolemic subjects. Metabolism.

[bib20] Krause J.-P., Schwenke K.D. (2001). Behaviour of a protein isolate from rapeseed (*Brassica napus*) and its main protein components — globulin and albumin — at air/solution and solid interfaces, and in emulsions. Colloids Surf. B Biointerfaces.

[bib21] Ladjal-Ettoumi Y., Boudries H., Chibane M., Romero A. (2016). Pea, chickpea and lentil protein isolates: physicochemical characterization and emulsifying properties. Food Biophys..

[bib22] Laemmli U.K. (1970). Cleavage of structural proteins during the assembly of the head of bacteriophage T4. Nat..

[bib23] Lawrence A.-M., Besir H. (2009). Staining of proteins in gels with Coomassie G-250 without organic solvent and acetic acid. J. Vis. Exp..

[bib24] Li N., Qi G., Sun X.S., Wang D., Bean S., Blackwell D. (2014). Isolation and characterization of protein fractions isolated from camelina meal. ASABE.

[bib25] Lopez C., Novales B., Rabesona H., Weber M., Chardot T., Anton M. (2021). Deciphering the properties of hemp seed oil bodies for food applications: lipid composition, microstructure, surface properties and physical stability. Food Res. Int..

[bib26] Lopez C., Rabesona H., Novales B., Weber M., Anton M. (2023). Walnut (Juglans regia L.) kernel oil bodies recovered by aqueous extraction for utilization as ingredient in food emulsions: exploration of their microstructure, composition and the effects of homogenization, pH, and salt ions on their physical stability. Food Res. Int..

[bib27] Lopez C., Sotin H., Rabesona H., Novales B., Le Quéré J.-M., Froissard M., Faure J.-D., Guyot S., Anton M. (2023). Oil bodies from chia (salvia hispanica L.) and camelina (camelina sativa L.) seeds for innovative food applications: microstructure, composition and physical stability. Foods.

[bib28] Manninen S., Lankinen M., Erkkilä A., Nguyen S.D., Ruuth M., de Mello V., Öörni K., Schwab U. (2019). The effect of intakes of fish and Camelina sativa oil on atherogenic and anti-atherogenic functions of LDL and HDL particles: a randomized controlled trial. Atherosclerosis.

[bib29] Matteo R., Pagnotta E., Ugolini L., Righetti L., Tavarini S., Lazzeri L., Farooq M., Siddique K.H.M. (2023). Neglected and Underutilized Crops.

[bib30] McClements D.J., Grossmann L. (2021). A brief review of the science behind the design of healthy and sustainable plant-based foods. npj Sci. Food.

[bib31] Mondor M., Hernández-Álvarez A.J. (2022). Camelina sativa composition, attributes, and applications: a review. Eur. J. Lipid Sci. Technol..

[bib32] Neupane D., Lohaus R.H., Solomon J.K.Q., Cushman J.C. (2022). Realizing the potential of camelina sativa as a bioenergy crop for a changing global climate. Plants.

[bib33] Ngo N.T.T., Shahidi F. (2021). Functional properties of protein isolates from camelina (Camelina sativa (L.) Crantz) and flixweed (sophia, Descurainis sophia L.) seed meals. Food Prod., ProceSs.Nutr..

[bib34] Nikiforidis C.V. (2019). Structure and functions of oleosomes (oil bodies). Adv. Colloid Interface Sci..

[bib35] Nikiforidis C.V., Kiosseoglou V. (2009). Aqueous extraction of oil bodies from maize germ (Zea mays) and characterization of the resulting natural oil-in-water emulsion. J. Agric. Food Chem..

[bib36] Nikiforidis C.V., Matsakidou A., Kiosseoglou V. (2014). Composition, properties and potential food applications of natural emulsions and cream materials based on oil bodies. RSC Adv..

[bib37] Ntone E., van Wesel T., Sagis L.M.C., Meinders M., Bitter J.H., Nikiforidis C.V. (2021). Adsorption of rapeseed proteins at oil/water interfaces. Janus-like napins dominate the interface. J. Colloid Interface Sci..

[bib38] Ostrikov A.N., Kleimenova N.L., Kopylov M.V., Bolgova I.N. (2021). The study of the fatty acid composition of camelina oil obtained by cold pressing. IOP Conf. Ser. Earth Environ. Sci..

[bib39] Puglisi M.J., Fernandez M.L. (2022). The health benefits of egg protein. Nutr..

[bib40] Righini D., Zanetti F., Monti A. (2016). The bio-based economy can serve as the springboard for camelina and crambe to quit the limbo. OCL.

[bib41] Sydor M., Kurasiak-Popowska D., Stuper-Szablewska K., Rogoziński T. (2022). *Camelina sativa*. Status quo and future perspectives. Ind. Crop. Prod..

[bib42] Tavarini S., De Leo M., Matteo R., Lazzeri L., Braca A., Angelini L.G. (2021). Flaxseed and camelina meals as potential sources of health-beneficial compounds. Plants.

[bib43] Tzen J., Cao Y., Laurent P., Ratnayake C., Huang A. (1993). Lipids, proteins, and structure of seed oil bodies from diverse species. Plant Physiol..

[bib44] Veljković V.B., Kostić M.D., Stamenković O.S. (2022). Camelina seed harvesting, storing, pretreating, and processing to recover oil: a review. Ind. Crop. Prod..

[bib45] Wanasundara J.P.D. (2011). Proteins of Brassicaceae oilseeds and their potential as a plant protein source. Crit. Rev. Food Sci. Nutr..

[bib46] Zanetti F., Alberghini B., Marjanović Jeromela A., Grahovac N., Rajković D., Kiprovski B., Monti A. (2021). Camelina, an ancient oilseed crop actively contributing to the rural renaissance in Europe. A Rev. Agron. Sustain. Dev..

[bib47] Zubr J. (2003). Dietary fatty acids and amino acids of camelina sativa seed. J. Food Qual..

